# Erector Spinae Plane Block as an Armamentarium for Pain Management in a Pyeloplasty Patient With Arrhythmogenic Right Ventricular Cardiomyopathy: A Case Report

**DOI:** 10.7759/cureus.67724

**Published:** 2024-08-25

**Authors:** Sandeep Veer, Abhishek Raj, Ram Prakash B U, Amala Kale

**Affiliations:** 1 Anaesthesiology, Dr. D. Y. Patil Medical College, Hospital and Research Centre, Dr. D. Y. Patil Vidyapeeth (Deemed to be University), Pune, IND; 2 Community Medicine, Dr. D. Y. Patil Medical College, Hospital and Research Centre, Dr. D. Y. Patil Vidyapeeth (Deemed to be University), Pune, IND

**Keywords:** sugammedex, enhanced recovery after surgery, erector spinae block, multimodal analgesia, arrhythmogenic right ventricular cardiomyopathy

## Abstract

This case report presents the anaesthesia management of a 21-year-old male with arrhythmogenic right ventricular cardiomyopathy (ARVC) undergoing pyeloplasty. An erector spinae plane block (ESPB) was employed as part of a multimodal analgesia approach to minimize intraoperative stress and reduce opioid consumption. The ESPB was administered using 20 mL of 0.5% ropivacaine with clonidine under ultrasound guidance, providing effective somatic and visceral analgesia. The intraoperative period was uneventful, and the patient had a stable postoperative recovery. This case highlights the potential of ESPB as a safe and effective anaesthesia technique in patients with ARVC, offering enhanced postoperative pain management and aligning with Enhanced Recovery After Surgery (ERAS) protocols.

## Introduction

Arrhythmogenic right ventricular dysplasia/cardiomyopathy (ARVD/C) is a genetic heart disorder characterized by the replacement of right ventricular muscle cells with fibrofatty tissue, resulting in electrical instability and an elevated risk of ventricular tachycardia (VT) [1]. An erector spinae plane block (ESPB) is a regional anaesthesia technique used for pain management. It involves the injection of a local anaesthetic into the plane between the erector spinae muscle and the transverse processes of the vertebrae. ESPB is an alternative to neuraxial block for post-surgical pain in renal surgeries [2]. Despite the challenges in diagnosing and managing patients with ARVC, we report a case of successful anaesthesia management. We were able to navigate the procedure without encountering any lethal arrhythmias, despite the inherent difficulties posed by this condition. Danish surgeon Dr. Kehler developed Enhanced Recovery After Surgery (ERAS) protocols for enhanced surgical recovery, now adopted across various specialities. The core principles are a patient-centred, evidence-based, multimodal approach. Various components include preoperative optimization of the patient, standardized anaesthesia, analgesia, stress reduction during the intraoperative period, early mobilization, and effective pain management in the postoperative period [3].

## Case presentation

**Figure 1:**
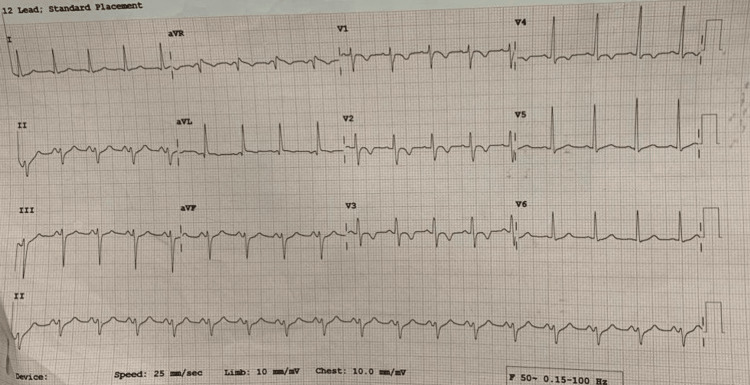
Preoperative electrocardiogram Preoperative electrocardiogram showing left axis deviation, sinus tachycardia, and T-wave inversion in leads V2, V3, and V4.

A 21-year-old male patient, a known case of ARVC for four months, was posted for right-sided pyeloplasty under general anaesthesia. The patient was diagnosed with ARVC four months ago after experiencing five episodes of seizures accompanied by loss of consciousness. Since the diagnosis, the patient has been on propranolol 5 mg twice daily. His electrocardiogram showed left axis deviation, sinus tachycardia, and T-wave inversion in leads V2, V3, and V4, as shown in Figure 1. Preoperative 2D echogram showed a dilated right atrium, dilated right ventricle, dilated right ventricular outflow tract, mild tricuspid regurgitation, right ventricular fractional area change <30%, RV apical dyskinesia, and ejection fraction of 60%.

On the day of surgery, the patient’s vital signs in the operating room were recorded as BP 140/100 mmHg, HR 110/min, and SpO₂ 100%. After confirming the morning dose of 5 mg propranolol and obtaining written informed consent, the patient was transferred to the operating theatre (OT) with all emergency cardiac drugs and an external defibrillator ready. The patient was then premedicated with midazolam (0.02 mg/kg), followed by the induction of general anaesthesia with fentanyl (2 µg/kg) and etomidate (0.5 mg/kg). Tracheal intubation was done with an 8-size endotracheal tube after the administration of vecuronium. To reduce the stress response, intubation was done by a senior anaesthetist, and 2% lignocaine spray was used to anaesthetise the vocal cords just before intubation.

**Figure 2:**
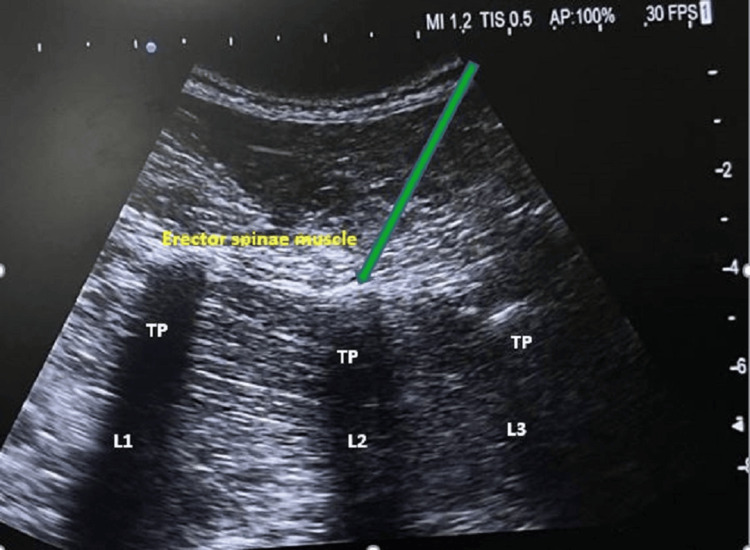
Intraoperative ultrasonography guided erector spinae block TP: Transverse process; L1: Lumbar vertebrae 1; L2: Lumbar vertebrae 2; L3: Lumbar vertebrae 3

Anaesthesia was maintained by vecuronium for muscle relaxation and isoflurane as an inhalational agent. ETCO₂ was maintained in the range of 32 to 38 mmHg. An intraoperative arterial line was secured to monitor intra-arterial blood pressure. A multimodal analgesia technique was used to maximize pain relief while minimizing the side effects associated with any single modality. It also improves postoperative outcomes and provides better pain control [4]. After positioning, a right-sided ESPB was given at the second lumbar transverse process with a low-frequency 5-10 MHz curvilinear probe to reduce the surgical stress response, with 20 mL of 0.5% ropivacaine and 20 µg clonidine, as shown in Figure 2.

The intraoperative period was uneventful, with vitals: BP 104/61 mmHg, HR 71/min, mean arterial pressure (MAP) 80 mmHg, and SpO₂ 100%, and the surgery was completed as scheduled.

Intravenous lignocaine 2 cc (40 mg) was given to avoid extubation response. Once spontaneous respiration was established, extubation was done after decurarisation with injection sugammadex uneventfully. After extubation, the vitals were stable.

## Discussion

ARVC is a condition that affects about 1 in 3,000 individuals, with men being more frequently and earlier affected than women [1]. Common symptoms of ARVC include palpitations, light-headedness, and syncope. Most individuals with ARVC can present with chest pain associated with transient ischemic changes on the electrocardiogram and elevated troponin levels, which mimic the clinical presentation of myocardial infarction [5].

ARVC is characterized by diffuse or segmental loss of right ventricular myocytes, replaced by fibrofatty tissue, resulting in the thinning of the right ventricular wall [6]. This condition commonly presents in individuals aged 10 to 50 years. Diagnostic tools for ARVC include electrocardiogram, 2D echocardiography, angiography, computed tomography, and magnetic resonance imaging [7]. Treatment options primarily consist of antiarrhythmic agents, catheter ablation, diuretics, beta-blockers, and heart transplantation [8].

The ESPB offers several advantages, such as potentially reduced opioid consumption, improved postoperative pain control, and a lower risk of complications associated with systemic opioids or other analgesics [9]. It is essential for avoiding respiratory complications and ensuring better patient satisfaction. Typically performed using an in-plane ultrasound-guided technique, the anaesthetic drug spreads cephalad and caudad within the ESPB, blocking the dorsal and ventral rami of spinal nerves and the sympathetic chain, providing both somatic and visceral analgesia. This makes ESPB particularly effective for surgeries involving the thoracic and abdominal regions, where both visceral and somatic pain pathways are involved [10].

However, ESPB carries potential risks and complications, such as local anaesthetic toxicity, nerve injury, or infection. Proper technique, patient selection, and monitoring are essential to minimize these risks. ERAS protocols enhance surgical outcomes through comprehensive perioperative care strategies. Multimodal analgesia in ERAS includes various combinations of opioids, non-steroidal anti-inflammatory drugs (NSAIDs), local anaesthetics, wound infiltration, and nerve blocks. Ultrasound-guided nerve blocks are transforming perioperative care, enhancing patient satisfaction and recovery, and leading to significant reductions in postoperative pain scores, opioid use, and hospital stay [11].

## Conclusions

ARVC is a complex and formidable condition, characterized by its insidious progression, unpredictable arrhythmias, and propensity to affect young patients. The ESPB has emerged as a promising part of anaesthesia adjunctive therapy for ARVC, offering a novel intraoperative approach to managing this challenging condition. The primary benefit of ESPB in ARVC is the reduction of pain during surgical procedures, which can trigger tachycardia and stress responses on the ventricular walls. By abolishing pain and tachycardia, ESPB prevents ventricular stress, thereby reducing the risk of ARVC exacerbation.

Furthermore, ESPB facilitates enhanced postanaesthesia recovery and postoperative pain management, contributing to improved patient outcomes. The integration of ESPB into the multidisciplinary management of ARVC may improve treatment efficacy, reduce complications, and enhance the quality of life for affected individuals.
